# Building a multipurpose insertional mutant library for forward and reverse genetics in *Chlamydomonas*

**DOI:** 10.1186/s13007-017-0183-5

**Published:** 2017-05-15

**Authors:** Xi Cheng, Gai Liu, Wenting Ke, Lijuan Zhao, Bo Lv, Xiaocui Ma, Nannan Xu, Xiaoling Xia, Xuan Deng, Chunlei Zheng, Kaiyao Huang

**Affiliations:** 1 Key Laboratory of Algal Biology, Institute of Hydrobiology, Chinese Academy of Sciences, Wuhan, 430072 China; 2 University of Chinese Academy of Sciences, Beijing, 100039 China; 3 College of Life Sciences, Hubei University, Wuhan, 430062 China

**Keywords:** *Chlamydomonas*, Insertional mutants, Mutant library, Flagella, Intraflagellar transport, Oil droplet

## Abstract

**Background:**

The unicellular green alga, *Chlamydomonas reinhardtii*, is a classic model for studying flagella and biofuel. However, precise gene editing, such as Clustered Regularly Interspaced Short Palindromic Repeats (CRISPR) and CRISPR-associated protein (Cas9) system, is not widely used in this organism. Screening of random insertional mutant libraries by polymerase chain reaction provides an alternate strategy to obtain null mutants of individual gene. But building, screening, and maintaining such a library was time-consuming and expensive.

**Results:**

By selecting a suitable parental strain, keeping individual mutants using the agar plate, and designing an insertion cassette-specific primer for library screening, we successfully generated and maintained ~150,000 insertional mutants of *Chlamydomonas*, which was used for both reverse and forward genetics analysis. We obtained 26 individual mutants corresponding to 20 genes and identified 967 motility-defect mutants including 10 mutants with defective accumulation of intraflagellar transport complex at the basal body. We also obtained 929 mutants defective in oil droplet assembly after nitrogen deprivation. Furthermore, a new insertion cassette with splicing donor sequences at both ends was also constructed, which increased the efficiency of gene interruption.

**Conclusion:**

In summary, this library provides a multifunctional platform both for obtaining mutants of interested genes and for screening of mutants with specific phenotype.

**Electronic supplementary material:**

The online version of this article (doi:10.1186/s13007-017-0183-5) contains supplementary material, which is available to authorized users.

## Background

*Chlamydomonas reinhardtii* is a unicellular green alga that shares organelles with both higher plants and animals [1]. This organism performs photosynthesis via cup-shaped chloroplasts, moves toward nutrients and light using a pair of flagella/cilia, and accumulates oil droplets in response to nitrogen starvation [2]. So *Chlamydomonas* is recognized as an important model for studying photosynthesis, flagella/cilia, and stress response [3, 4]. Recently, It has been acknowledged as “green yeast” for the study of biofuels such as biodiesel and hydrogen, and has been used as a biofactory for the production of antibodies and biopharmaceuticals [5, 6].

Classic and modern genetic manipulation techniques have been established in the last 60 years since Gilbert Morgan Smith isolated and provided the first *C. reinhardtii* strain to the scientific community [7]. Ultraviolet (UV) light [8], ethyl methanesulfonate (EMS) [9] and *N*-methyl-*N*′-nitro-*N*-nitrosoguanidine (MNNG) [10, 11] was widely used for mutagenesis and generated many useful mutants. For example, the genes involved in photosystem-II and photosynthetic electron transport were first characterized in Paul Levine’s laboratory by screening UV-induced acetate auxotrophy mutants [8, 12]. Cell wall-deficient mutants have been generated by MNNG mutagenesis and isolated under stereomicroscopy. These mutants have been widely used for gene transformation using glass beads [13]. Remarkably, temperature-sensitive flagellar mutants generated by MNNG mutagenesis are motile at permissive temperatures, but become immotile at restrictive temperatures, and have been used to identify the components of intraflagellar transport (IFT) [11, 14].

Transformation or introducing a foreign gene in the genome is a prerequisite of modern genetics. Several methods have been developed to transform DNA fragments into the nuclear genome of *Chlamydomonas*. In addition to glass beads mentioned above, other techniques include electroporation, biolistic transformation and biotransformation by *Agrobacterium tumefaciens* are also used in *Chlamydomonas* [15–17]. Biolistic transformation can also be used to transform DNA into the chloroplast and mitochondria of *Chlamydomonas* [18, 19]. Meanwhile, various transformation markers have been developed. These include auxotrophic markers such as argininosuccinate lyase (*ARG7*) and nitrate reductase (*NIT1*), which encode enzymes that complement metabolic deficiencies [15, 20], as well as antibiotic markers such as *aphVII*, *aphVIII*, and *ble*, which provide resistance to hygromycin, paromomycin and zeocin respectively [21–23]. Several novel markers have also been developed recently, such as herbicide resistance genes glyphosate acetyltransferase (*GAT*), protoporphyrinogen oxidase (*PPO*), and phytoene desaturase (*PDS*) and codon-optimized kanamycin-resistance gene *nptII* and tetracycline resistance gene *tetX* [24–26].

Similar to other classical organism models, both forward and reverse genetics strategies are widely employed in the research of *Chlamydomonas*. Forward genetics study generally composed of four major steps: mutagenesis, phenotypic screening, mutation loci (gene) mapping, and phenotype rescue. Much progress has been made to optimize these four steps. At present, mutagenesis introduced by insertion of a DNA cassette into the genome is preferred [27–29]. The mutated gene can be identified using PCR map-based cloning, and whole genome sequencing [29–31]. The valuable *Chlamydomonas* mutant library screened by forward genetics could also be cryopreserved for further analysis [32]. Specific mutated phenotypes can be screened using high throughput methods such as fluorescence-activated cell sorting [33]. Phenotype rescue is relatively difficult, because the expressions of foreign genes are relatively low in *Chlamydomonas* [34]. However, this problem may be solved partially by modification of transgene sequences, such as codon optimization, incorporation of native promoters and introns, co-expression with antibiotic gene or luciferase, and use of a host strain defective in transgene silencing [34–36].

Several methods are also available for reverse genetic studies in *Chlamydomonas*. Target genes can be knocked down by using small interfering RNAs [37] and artificial miRNAs [38, 39]. Conditional knockdown of *Chlamydomonas* genes can be achieved by using inducible promoters such as nitrate-induced promoter *NIT1*, nickel-induced promoter *CYC6*, and CO_2_-induced promoter *CAH1* [40, 41]. However, the knockdown effects of RNAi [37] and artificial miRNAs [38] varies depending on genes and may disappear after several months. Gene knockout based on homologous recombination (HR) provides a great opportunity to study the function of non-lethal genes. Of note, is that the natural HR rate in *Chlamydomonas* is extremely low [42]. Several strategies have been described to increase the HR rate, such as use of single-stranded DNA, use of HR constructs coding an antibiotic marker in frame with the target gene, and induction of DNA breaks in the target gene via zinc-finger nucleases [42, 43]. However, these methods are either limited to special genes or the procedures are tedious. Recently, gene editing using CRISPR and Cas9 has been reported in *Chlamydomonas*, but the efficiency is too low to be applied to any genes, especially when the phenotypes of mutated genes are unknown [44–46]. Thus, constructing a random insertional mutant library provides an alternative method to obtain the mutants of specific genes. This strategy has been successfully applied to construct a single-use insertion mutant library and obtained 45 individual mutants representing 37 different genes by screening the approximately 100,000 insertional mutants with PCR [29]. Most recently, an indexed, mapped mutant library was developed based on the high-throughput approach, in which the mutants were transferred by highly automatic robots and cryopreserved in liquid nitrogen. The insertion sites were determined by deep sequencing methods, *Chlamydomonas Mme*I-based insertion site Sequencing (ChlaMmeSeq) and Linear and Exponential Amplification of insertion site sequence coupled with Paired-end Sequencing (LEAP-Seq) [27, 47]. As a results, 37,000 mutants covering 73% of the *Chlamydomonas* genome were identified based on a deconvolution method [47]. However, construction and maintenance of such a library requires automatic robot technology, which is expensive for a small group. The library is mostly used for reverse genetics and more than half of the insertions lie in the untranslated region (UTR) [47]. Therefore, the need for improvement in construction, maintenance, and screening the library still remains.

In this study, a library of approximately 150,000 *Chlamydomonas* transformants was constructed and maintained with optimized procedures. Both reverse and forward genetics were performed to identify mutants of specific genes or mutants with specific phenotypes. We obtained 967 mutants with defective motility, 929 mutants with fewer oil droplets in response to nitrogen deprivation, and 26 individual mutants representing 20 different genes related to flagella, glycosylation, and starch metabolism. This library can also be used to screen mutants for any *Chlamydomonas* gene of interest or mutants with specific phenotype defects.

## Results

### Optimizing methods for construction of the insertional library

The genomic size of *Chlamydomonas* is about 111 Mb and the genome encodes approximately 17,000 genes according to the prediction in Joint Genome Institute (JGI) version 5.5 of *Chlamydomonas* genome [1]. Since the integration of DNA fragment in the genome of *Chlamydomonas* is random, the minimum number of insertional mutants is 111,000 if one insertional mutation locating within any 1 kb of genome is required. Taking into account that the average size of *Chlamydomonas* genes is approximately 6 kb, each gene can be hit about five times. However, to generate and maintain this quantity of mutants is challenging for an individual group. To optimize the use of this resource, we intended this library to be used not only for reverse genetics, but also forward genetics, such as screening mutants for flagella assembly and formation of oil droplets.

1. Selection of the parental strain

**Fig. 1:**
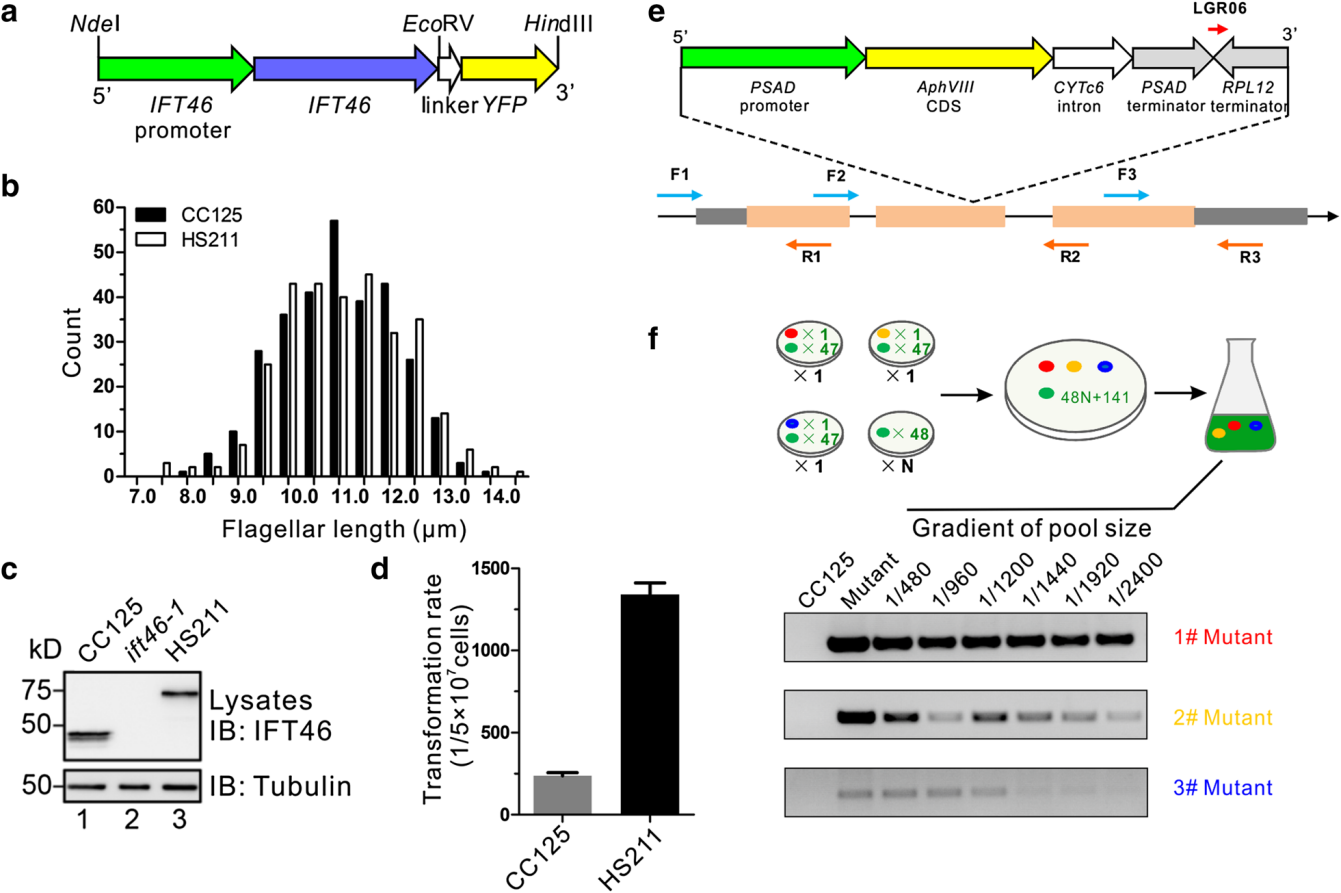
The optimized parental strain, primers, and size of the super pool for the insertional library. **a** The diagram shows the IFT46::YFP construct used to rescue the *ift46*-*1* mutant. **b** Flagellar length distribution in CC-125 and HS211 cells. **c** Western blot results demonstrated that IFT46::YFP was expressed in the HS211 strain. **d** Comparing the transformation efficiency of the HS211 strain with that of wild-type CC-125 cells. **e** The *aphVIII* cassette was used to generate the insertional mutation library; the *aphVIII* cassette-specific primer (LGR06), targeted gene-specific forward target primers (*F1*–*F3*), and reverse target primers (*R1*–*R3*) were designed to screen mutants for the target gene. **f** Determination of the maximal super pool size by PCR amplification of three known insertion loci in a series of super pools

Intraflagellar transport (IFT) plays a key role in flagellar assembly and disassembly [4]. In order to obtain the mutants defective in IFT protein accumulation at the basal body, we generated an IFT46::YFP fusion construct using an endogenous promoter of *IFT46* and transformed it into the flagella-less mutant *ift46*-*1* (Fig. 1a). IFT46::YFP rescued the motility defect of *ift46*-*1* and the distribution of flagellar length in the rescued strain was similar to the wild type, with an average length of 11.1 μm (Fig. 1b). The rescued strain was designated as HS211. Western blot results demonstrated that IFT46 was expressed in CC-125 cells only and not in the *ift46*-*1* mutant, while the fusion protein IFT46::YFP was detected in HS211 (Fig. 1c). These data suggest that the IFT46::YFP fusion protein functions as endogenous IFT46. Thus, HS211 could serve as a parental strain for screening of flagella assembly mutants and IFT basal body localization-defect mutants.

Next, we compared the transformation efficiency of HS211 with wild type strain. Since the *aphVII* gene conferring to hygromycin resistance was used to generate the HS211 strain, transformation efficiency of HS211 was tested using the aminoglycoside 3′-phosphotransferase (*aphVIII*) cassette as a selection marker. Using 5 × 10^7^ cells for one transformation, an average of 1342 transformants were obtained in HS211, whereas only 236 were obtained in CC-125 cells. The transformation efficiency of HS211 was sixfold higher than that of the wild type, CC-125 (Fig. 1d). This indicates that one transformation using 30 ng *aphVIII* in an electroporation cuvette generated almost 1400 transformants after 3 days, which suggests that only ~100 times transformations can generate enough mutants for construction of the library.

2. Insertion cassette and screening primer design

Besides serving as a selection marker for transformation, an ideal cassette for insertional mutagenesis can terminates transcription of the interrupted gene completely. Therefore, the 2.6 kb *Mly*I digested fragment from plasmid pMJ013b was used, which contains the promoter and terminator of *PSAD* gene, the *aphVIII* gene, an intron from *CYTc6*, and an *RBL12* terminator [27] (Fig. 1e). The intron was intended to enhance the expression of the *aphVIII* gene and two inverted terminators at the 3′UTR were designed to terminate gene transcription of interrupted gene, irrespective of the direction of insertion. The fragment was therefore, selected as the insertion cassette.

The principle behind PCR-based screening was that when an insertion occurred in a target gene, a PCR product can be obtained using an insertion cassette-specific primer and a target gene-specific primer [29]. Since GC content of the *Chlamydomonas* genome is approximately 60%, the PCR product should be <1 kb in order to be considered reliable. Therefore, the target gene-specific primers were designed in two directions, forward and reverse, at an interval of approximately 1.0 kb. In order to cover every possible insertion events, the target gene-specific primers were designed from 500 bp upstream of 5′UTR to the end of 3′UTR of any interested genes (Fig. 1e).

The insertion cassette-specific primer requires to be within the 200- to 500-bp region from the insertion cassette border [29]. This region not only ensures amplification specificity and efficiency, but also reduces the possibility of corruption of the annealing sites caused by recombination or endonucleolytic cleavage during transformation [27]. The primer previously used has been designed as per the *aphVIII* gene sequence or the endogenous sequence of the *PSAD* promoter and *RBL12* terminator. The *aphVIII* gene in this 2.6-kb cassette was approximately 700 bp from the border, while the primers annealing to *PSAD* promoter and *RBL12* terminator tended to generate non-specific amplification since there is another copy of these two genes in the genome. Therefore, we designed the cassette-specific primer LGR06, annealing to the junction between *PSAD* and *RBL*12 terminator, which was 273 bp near the border of insertion cassette. Compared with the cassette-specific primer LGL03, which anneals to the *PSAD* promoter, the sequence of LGR06 does not exist within the *Chlamydomonas* genome. PCR amplifications with LGR06 generate fewer non-specific products and a cleaner background using the library and HS211 genomic DNA as templates. This primer increased efficiency of library screening (Additional file 1: Fig. S1).

3. Determination of the super pool size

A super pool size up to approximately 1000 was adopted for mutants screening in the previously generated library [29]. Larger pool size can reduce labor cost significantly if PCR amplification sensitivity can be guaranteed. We identified the maximal size of the super pool for library screening by mixing three characterized insertional mutants with varying numbers of uncharacterized insertional mutants, to generate a series of super pools ranging from 480 to 2400 in size (Fig. 1f). PCR amplification of three different insertion loci indicated that two loci could be detected in super pool reached to 2, 400, another one only could be identified by PCR in super pool 1920, so we choose 1440 as the optimal size for super pool (Fig. 1f).

4. Maintenance and replication of transformants

**Fig. 2:**
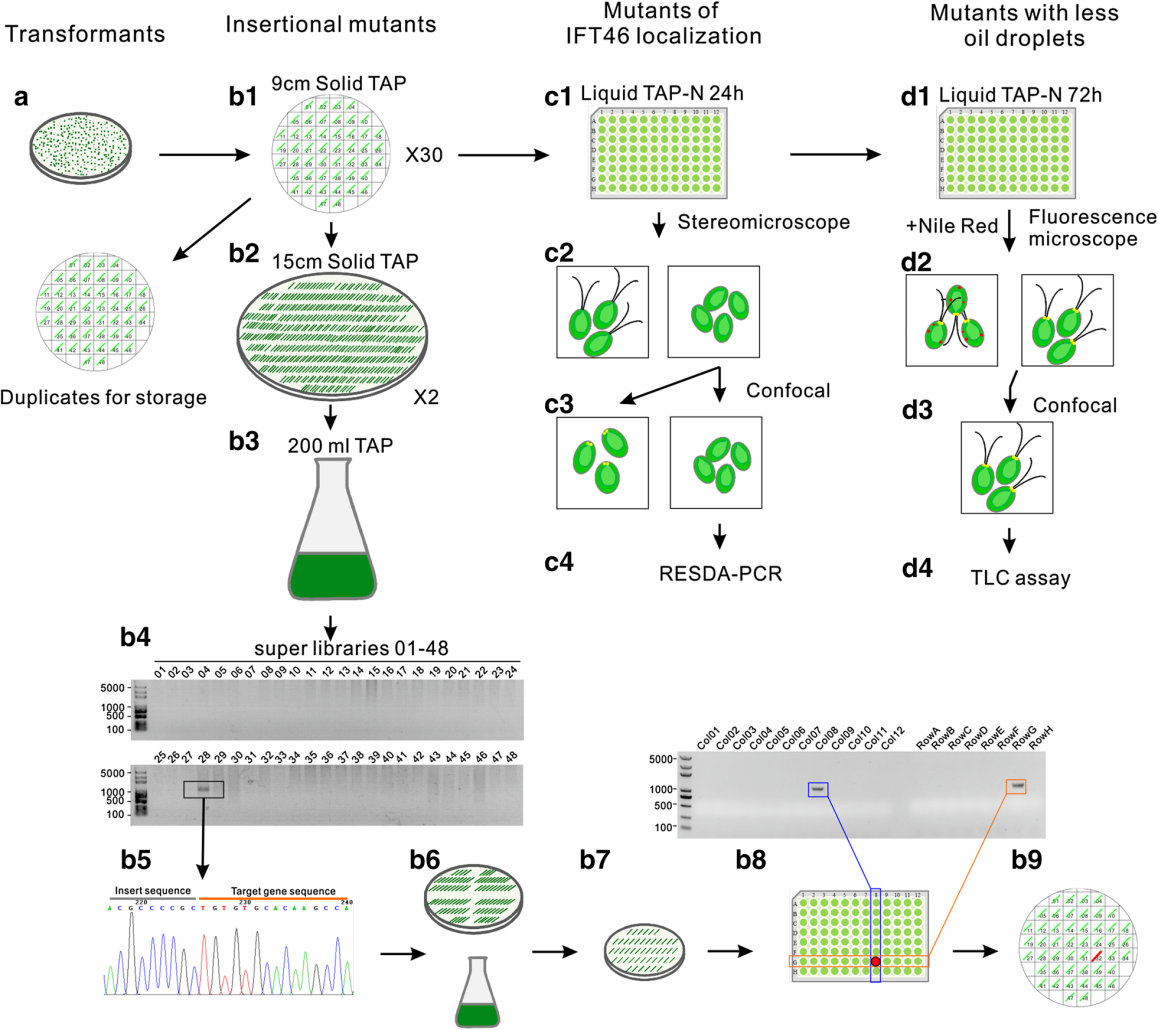
Screening of insertional mutants based on reverse and forward genetics. *a* Transformants were generated using HS211 as the parental strain and *aphVIII* as the insertion cassette. *b1* A total of 48 transformants were grown on a 9-cm solid TAP plate and a pair of plates comprised the basic pool of 96 transformants; all plates were duplicated every 4 months for long-term storage. *b2* A super pool was generated by replicating transformants from thirty 9-cm plates to two 15-cm plates. *b3* Cells grown in *b2* were resuspended in 200 ml TAP medium; genomic DNA was extracted and stored as super pool DNA libraries. *b4* Mutants were screened by PCR using insertion cassette-specific primer, LGR06, a target gene-specific primer and 48 super-pool DNA libraries as templates. *b5* PCR products were sequenced to confirm the insertion locus. *b6* DNA of basic pools from the positive super pool was extracted separately. *b7* The positive basic pool was identified by PCR using the specific target primer and the basic pool DNA library as templates. *b8* The cells were mixed together by *line and row* respectively, and the positive transformant was localized by colony PCR adopting a cross-over strategy. *b9* Positive transformants were recovered from the 9-cm plate for storage in *b1*. *c1* Cells from the basic pool were transferred to nitrogen-deprived TAP (TAP-N) medium for 24 h in microtitle plates for gametogenesis. *c2* Mutants with motility defects were identified under a stereomicroscope. *c3* Mutants of IFT46 localization were characterized using confocal microscopy. *c4* The putative insertional mutagenesis was identified using RESDA-PCR. *d1* Cells in *c1* are transferred into TAP-N medium for another 48 h, inducing the formation of oil droplets. *d2* After Nile *red* staining, the mutants with fewer oil droplets were identified with fluorescence microscopy. *d3* The phenotype with fewer oil droplets was confirmed under confocal microscopy. *d4* The quantity of TAG in mutants identified in D3 was analyzed by thin-layer chromatography (TLC)

Maintenance and replication of large-scale *Chlamydomonas* mutants is a time-consuming task. To overcome this problem, a single-use library of up to 100,000 transformants was generated [29]. Alternatively, a library consisting of 18,334 transformants was cultured in 96- or 384-μl plates and cryopreserved with automatic robots operated under highly sterile conditions [47]. We intended to save all transformants on an agar plate and therefore devised an optimal economic strategy. After electroporation, the transformants grew on solid *tris*–acetate–phosphate (TAP) plates under low light for 1 week (Fig. 2a), then each clone was picked up with a toothpick and applied along a line across the agar surface, where the clone propagated to the entire length of the line within a week. Thus, 48 lines were obtained (Fig. 2b1). This method not only guaranteed enough cell count for further analysis and reduced the duration for replication of the library, but also reduced loss of transformants due to contamination, since only 48 colonies would be lost in case a plate was contaminated. After a year, the livability of our library was approximately 97.8%, and the contamination rate was lower than 3%.

5. Isolation of the super pool DNA

Successful PCR amplification of insertion loci depends on quality of DNA templates from each super library. The library should contain sufficient DNA for thousands of PCR runs and also contain similar amount of genomic DNA of each mutant. To achieve this goal, we inoculated 1440 transformants from the basic pool onto 15-cm sized plates by drawing a shorter colony line (Fig. 2b2), such that the similar amount of cells from each clone could be guaranteed. All the transformants grown into visible clones of similar size within several days, then the clones were scraped and resuspended in liquid TAP medium, forming a cell culture with concentration >5 × 10^6^ cells/ml. After the cell reached stationary phase under constant light, genomic DNA was isolated using the phenol/chloroform method [48]. Generally, about 250 μg genomic DNA can be obtained from a single super pool library preparation, which is adequate to screen 2500 pairs of primers.

### Screening the mutants based on reverse genetics strategy

We planned to screen insertional mutants of 61 genes of interest, which requires more than 600 target gene-specific primers to screen 104 super pool libraries (Additional file 2: Table S1 and Additional file 3: Table S2). This means that at least 62,000 PCR reactions and 630 PCR runs need to be performed. To save time and cost, we optimized several aspects of the PCR screening strategy. First, the super pool libraries were divided into two groups, each including 48 libraries. If a reliable mutant was identified in one group, the other group was skipped. Second, DNA templates were arrayed into a matrix consisting of 4 rows and 12 columns. All PCR components were premixed and the reaction buffers were prepared only with addition of different target-specific primers. Thus, templates and buffers could be transferred into PCR microplates with a multichannel pipette (Fig. 2b4). Third, since it is generally accepted that insertional mutagenesis near the start codon tends to induce a complete knock-out effect, the screening process for each target gene was initiated with the first six target primers either forward or reverse from the 5′UTR end. If a correct insertion was identified by sequencing the PCR product, the other primers and libraries were not screened (Fig. 2b5). As a result, the total number of PCR runs was largely reduced. Finally, the volume of each PCR was reduced to 20 μl and the amount of Taq polymerase used per reaction was reduced from 2 units (supplier recommended) to 0.5 units, which improved amplification specificity per contra.

Once a correct insertion was identified from a super pool, the basic pools from this positive super pool were recovered for DNA isolation (Fig. 2b6). All 96 colonies from each basic pool were replicated onto a fresh 9-cm solid agar plate, allowing all transformants to recover to similar extents (Fig. 2b6). Then, cells from these plates were washed with 20 ml TAP medium and genomic DNA from each basic pool was isolated using the phenol/chloroform method, but on a small scale (Fig. 2b6). Approximately 10 μg basic library DNA was harvested, which was adequate for identification of positive transformants from the basic pool (Fig. 2b7). Once the positive basic pool was identified, all 96 transformants from the positive basic pool were inoculated separately, into liquid TAP medium contained in 96 microtiter plates. The cells were mixed together by line and row respectively, and colony PCR was performed. Positive results were obtained from both, the row and line mixtures as a positive transformant (Fig. 2b8). Finally, the positive transformant was duplicated from the original 9-cm solid agar plates for further analysis (Fig. 2b9).

### Screening flagellar assembly-defect mutants

**Fig. 3:**
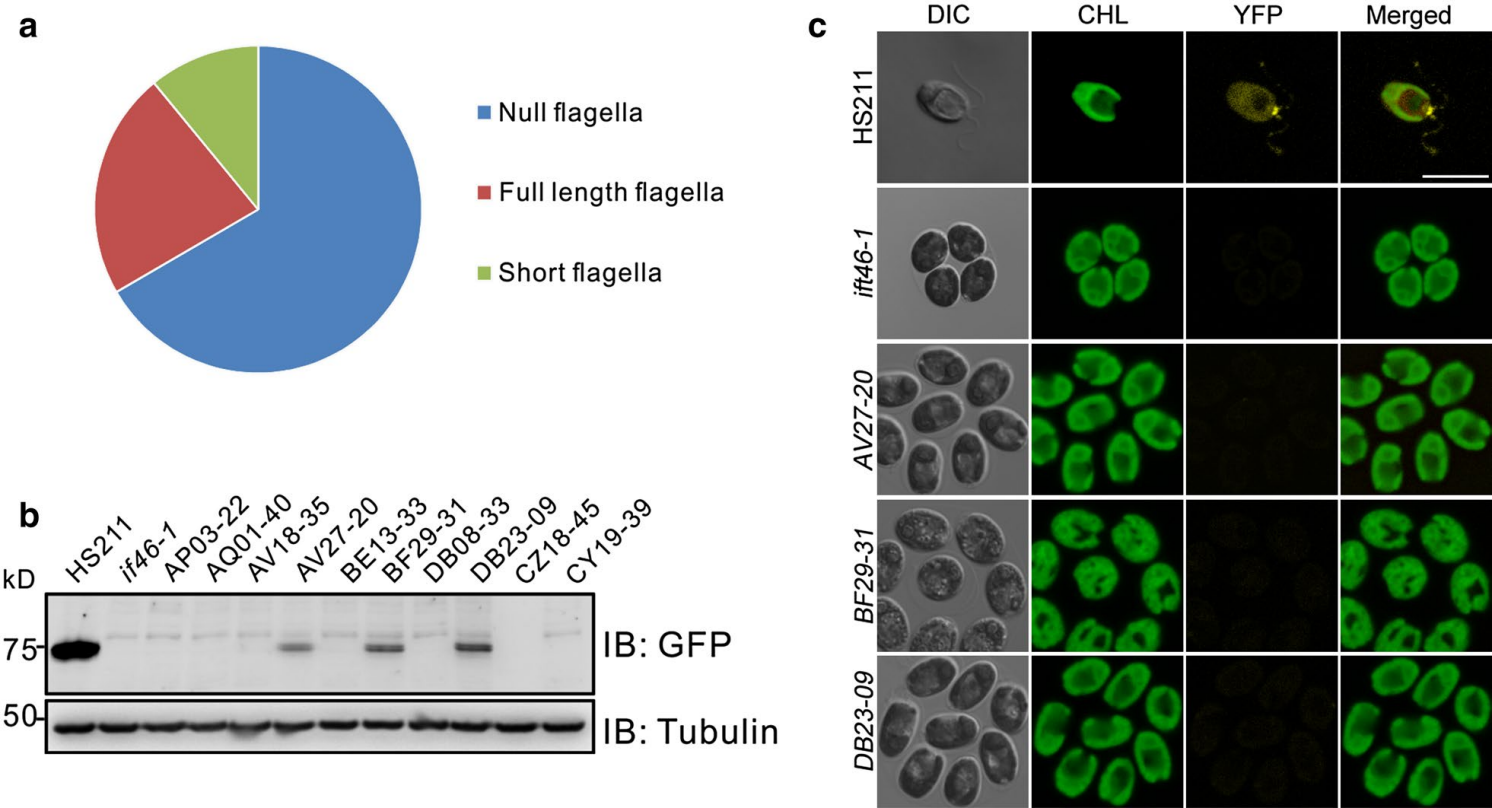
Mutants with motility defects and abnormal IFT46 localization. **a** Flagellar motility defects resulted from null, short, or paralyzed flagella. **b** Expression profile of IFT46::YFP in HS211 and the mutants. **c** Fluorescence micrographs showing IFT46::YFP localized at the basal body in HS211 cells and disappeared in mutants. *Scale bar* 10 μm. *CHL* chlorophyll fluorescence

In addition to screening mutants for any gene of interest by reverse genetics, the library was also utilized to screen flagellar-defect mutants. Since the flagella of gametes are longer than those of vegetative cells [49], an aliquot of each clone was transferred into liquid nitrogen-deprived TAP (TAP-N) medium in 96-well microtiter plates for gametogenesis and screened for mutants with motility defects under a stereomicroscope (Fig. 2c1). We identified 967 motility-defect mutants (Fig. 2c2). Since motility defects may result from flagella loss or from the flagella being short or paralyzed, the flagellar length of these mutants was measured. We found that 66% of these mutants failed to assemble flagella, 11% showed shorter flagella, while the rest exhibited flagella with normal length, but were paralyzed. This suggests that most motility defects are caused by aberrations in flagella assembly (Fig. 3a).

Because the IFT marker IFT46::YFP was introduced into the parental strain HS211, all motility-defect mutants with aberrant IFT distribution at the basal body were screened. In the wild type HS211, the puncta of IFT46::YFP were distributed along the flagella and IFT46::YFP accumulation was observed at the basal body (Fig. 3c). In most of 638 flagella-less mutants, IFT46::YFP was concentrated at the basal body, while the fluorescence signal from some of these mutants was a little weaker. However, ten mutants showed similar phenotype with the *ift46*-*1* mutant, lacking IFT46 accumulation at the basal body (Fig. 3c). The loss of IFT46::YFP signal was probably caused by knockout of IFT46::YFP in the parental strain, interference of IFT46 transcription, or regulation of IFT46 anchoring to basal body. To investigate this, IFT46::YFP expression in ten mutants was analyzed by western blot (Fig. 3b). IFT46::YFP proteins were absent in seven mutants, but were detected in the remaining three mutants, at reduced quantities compared to the parental strain HS211. This indicates that certain key elements regulating IFT46 anchorage to the basal body may have been knocked out in these three mutants. The flanking sequences of the insertion cassette in these mutants were amplified using restriction enzyme site-directed amplification (RESDA)-PCR in order to map the insertion loci (Table 1). In the mutant DB23-09, the cassette was inserted into the ninth intron of *IFT88*, which is required for the localization of IFT-B complex to the basal body [50]. The characterization of *ift88* mutant as exhibiting IFT46 distribution defect suggests that the screening strategy we used was reasonable. The other two insertion loci in mutants AV27-20 and BF29-31 were localized to genes Cre14.g633700, which is an uncharacterized protein with transmembrane domain, and Cre11.g467653, which is annotated as threonine-specific protein kinase. Nevertheless, further analyses are required to confirm their role in regulation of IFT46 accumulation at the basal body.

**Table 1:** The mutants lacking IFT46::YFP accumulation at the basal body

Strain	Accession of inserted gene^a^	Annotation	Insertion loci^b^
AP03-22	Cre11.g467717	Nitrilase-related; carbon–nitrogen hydrolase	R, 5th intron
AQ01-40	Not identified	–	–
AV18-35	Between Cre14.g612250 and Cre14.g612226	No annotation	Intergenic region
AV27-20	Cre14.g633700	Uncharacterized conserved protein	R, 4th intron
BE13-33	Not identified	–	–
BF29-31	Cre11.g467653	Threonine-specific protein kinase	R, 2nd intron
DB08-33	Not identified	–	–
DB23-09	Cre07.g335750	Intraflagellar transport protein 88	F, 9th intron
CZ18-45	Not identified	–	–
CY19-39	Cre12.g554400	No annotation	F, 1st exon

^a^Gene accession numbers were from Phytozome v10.3 *C. reinhardtii* website

^b^F and R indicate that the insertion cassettes have the same or opposite orientation as the targeted genes, respectively

### Screening mutants with defective in oil droplet assembly

**Fig. 4:**
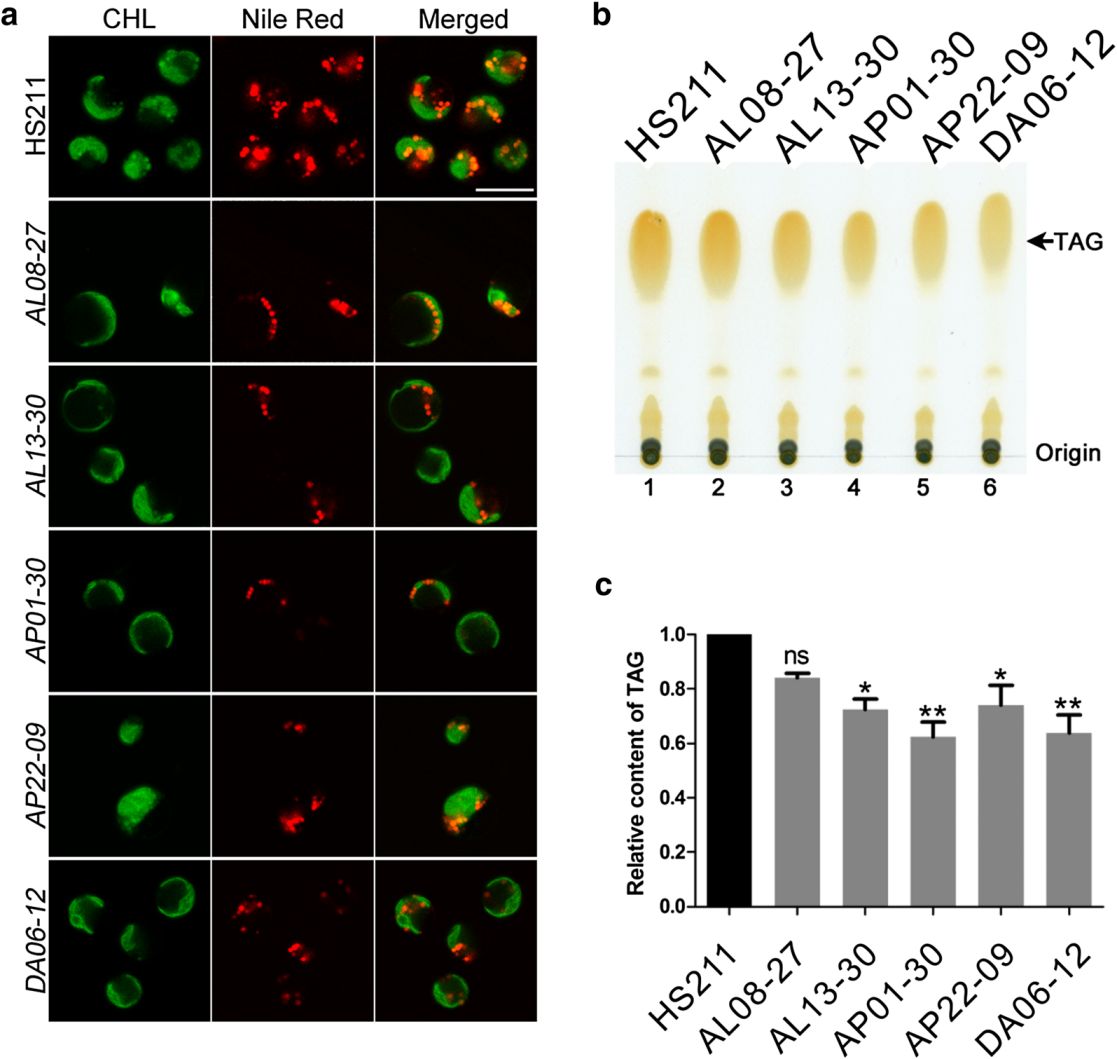
Mutants with defects in oil droplet formation and TAG synthesis. **a** Nile *red* staining of oil droplets in the mutants and HS211 after 3-day nitrogen starvation. *Scale bars* 10 μm. *CHL* chlorophyll fluorescence. **b** Analysis of TAG biosynthesis in mutants with defective oil droplet formation. **c** The relative amount of TAG in the mutants as compared to that in WT. *p < 0.05; **p < 0.01

*Chlamydomonas* accumulates oil droplets in response to nitrogen deprivation [51]. Therefore, we used this library to screen oil droplet assembly mutants. The parental strains and mutants were cultured in TAP-N medium for 48 h, and stained with Nile red. Oil droplets accumulated in the cell body were observed under a fluorescence microscope (Fig. 2d1). A total of 929 mutants with fewer oil droplets were characterized (Fig. 2d2); of these, 11 mutants were selected and observed under a confocal microscope (Fig. 2d3). The fluorescence intensities of Nile red in 5 mutants were decreased significantly as compared to the parental strain, representing a reduced number and size of oil droplets (Fig. 4a). Thin layer chromatography (TLC) indicated that 4 mutants synthesized less triacylglycerol (TAG) after nitrogen deprivation (Fig. 4b, c). The mutant of AP01-30 and DA06-10 only have 60% TAG compared the parental strains HS211. These data suggest that a large amount of mutants possessing defects in oil droplet formation have been identified in this study, which is a great resource to study the molecular mechanism for assembly and disassembly of oil droplets.

### Characterization of mutants for targeted genes

**Fig. 5:**
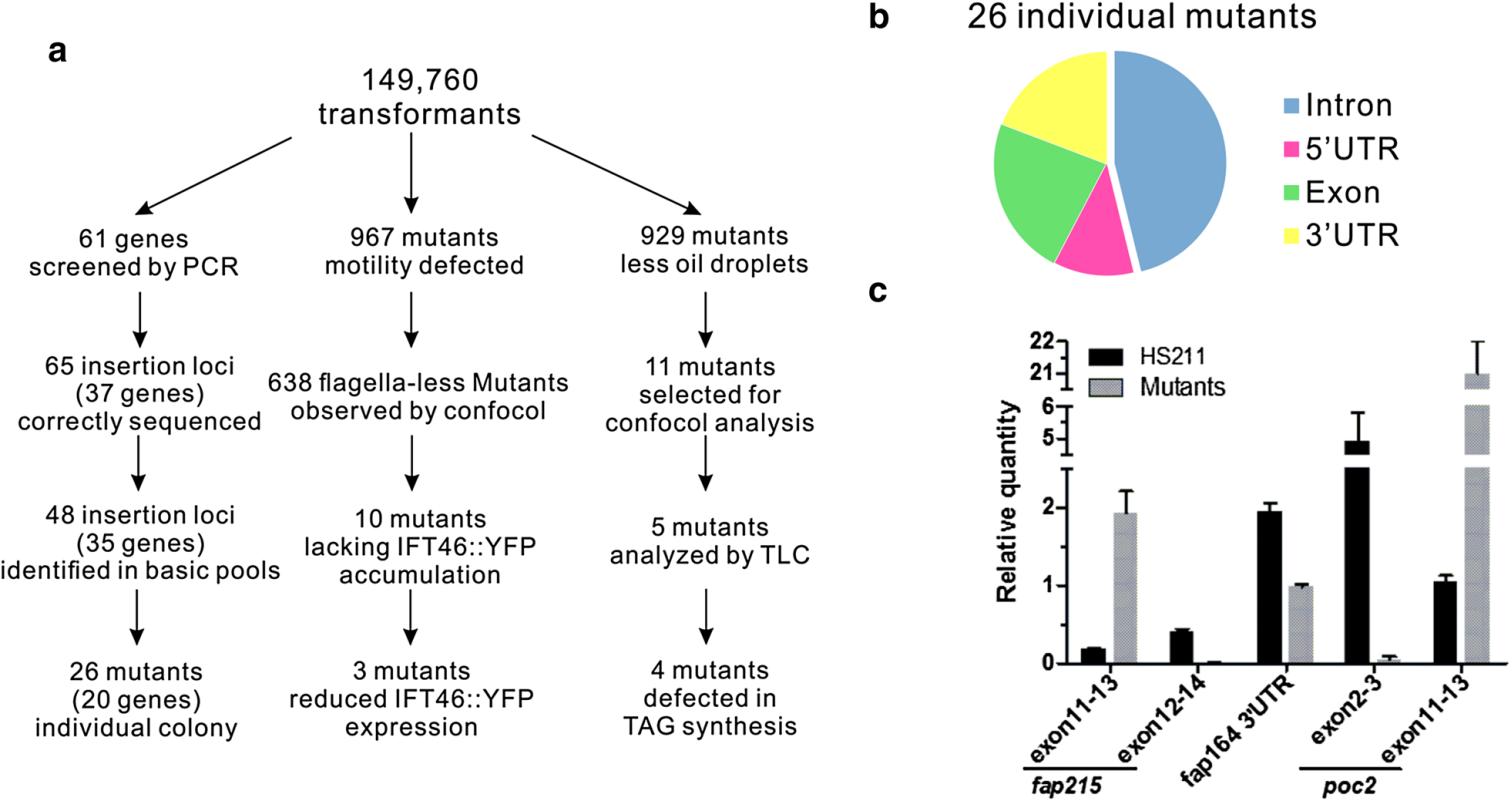
Summary of identified mutants. **a** Number of transformants identified at each steps during screening. **b** Distribution of insertion loci in the mutants. **c** Expression levels of target genes in which an insertion cassette was inserted in an intron

Initially, we planned to screen 61 mutants for genes involved in flagellar assembly, biogenesis of basal body, and vesicles trafficking (Additional file 2: Table S1). We identified 65 correct insertion loci corresponding to 37 genes from the super pool library during 6 months. Due to contamination and low recovery rate of certain transformants, only 48 insertion loci could be amplified in the basic pool. Finally, 26 mutants corresponding to 20 target genes were identified, including transition zone mutant *POC2*, flagella mutant *PKD2*, glycosylation mutant *MAN1*, and starch metabolism mutant *SSS2* (Fig. 5a; Table 2). Among them, 31% insertion cassettes were integrated in the UTR, 23% were integrated in exons, and approximately 46% of insertions occurred in the introns (Fig. 5b). To determine whether mutagenesis in introns could induce complete knockout of target genes in *Chlamydomonas*, we compared the mRNA level of target genes in three individual mutants with parental strain HS211 by quantitative PCR, with probes locating at upstream, downstream, or across the insertion loci. The transcriptions of the three targeted genes were still detectable in the mutants. However, the transcript fragments upstream of the insertion loci (inserted in 13th intron) in mutant *fap215* were up-regulated. In contrast, the transcript across the insertion loci in mutant *fap215* and *poc2* (inserted in 2nd intron) was undetectable. The transcription downstream of the insertion loci was variable, which was down-regulated in mutant *fap164* (inserted in 2nd intron), but up-regulated by 20-fold in mutant *poc2* (Fig. 5c). Partial transcription was still detectable in the three mutants, which suggests that the 2.6-kb *Mly*I-digested expression cassette did not always terminate gene transcription completely when it was inserted into the intron. Therefore, another optimized insertion cassette was designed.

**Table 2:** List of identified mutants

Targetedgenes	Accession^a^	Description	Function category	Insertion loci^b^
*POC2*	Cre10.g436000	Polycystin-related	Transition zone	R, 2nd intron; F, 1st exon
*CRX*	Cre03.g202950	Ef-hand calcium-binding domain containing protein	Calredoxin	R, 2nd intron
*MAN1*	Cre07.g336600	Alpha-1,2-mannosidase	Glycosylation	R, 7th exon
*CUL3*	Cre17.g734400	Ubiquitin ligase SCF complex subunit Cullin	Ubiquitin ligase	R, 3′UTR
*XYLT*	Cre09.g391282	Glycoprotein 2-beta-d-xylosyltransferase	Glycosylation	F, 6th intron; F, 7th intron
*PKD2*	Cre17.g715300	Polycystin cation channel protein 2	Flagellar protein	F, 11th exon; F, 3rd exon
*AMYB1*	Cre06.g307150	Beta-amylase	Starch metabolism	R, 5′UTR; R, 3′UTR (two different mutants)
*SSS2*	Cre03.g185250	Soluble starch synthase II	Starch metabolism	R, 1st intron
*FAP215*	Cre03.g180450	5′-nucleotidase and flagellar associated protein	Flagellar protein	R, 13th intron
*FAP208*	Cre11.g482001	Ankyrin repeat-containing protein	Flagellar protein	F, 22nd exon
*FAP249*	Cre17.g746697	Flagellar associated protein	Flagellar protein	R, 5′UTR (ligated with 480 bp unknown sequence)
*FAP164*	Cre17.g735350	Flagellar associated protein	Flagellar protein	F, 2nd intron
*CDPK3*	Cre01.g009500	Protein kinase domain (Pkinase)	Flagellar protein	F, 2nd intron (ligated with partial left border of insertion fragments)
*PDCD6*	Cre03.g200050	Ca2+ -binding protein, EF-hand protein superfamily	ESCRT	R, 2nd intron; R, 4th intron
*VPS4*	Cre02.g079300	AAA-ATPase of VPS4/SKD1 family	ESCRT	R, 3′UTR
*VPS37*	Cre08.g362550	Subunit of the ESCRT-I complex	ESCRT	F, 2nd intron
*PEPC2*	Cre03.g171950	Phosphoenolpyruvate carboxylase	Lipid metabolism	R, 19th exon
*IFT139*	Cre06.g268800	Intraflagellar transport protein 139	IFT	R, 22nd intron
*UBA1*	Cre09.g386400	Ubiquitin-activating enzyme E1	Ubiquitin	R, 3′UTR
*FRE2*	Cre05.g241400	Ferric-chelate reductase (NADH)	Ferric protein	R, 5′UTR

^a^Gene accession numbers were from Phytozome v10.3 *C. reinhardtii* website

^b^F and R indicate that the insertion cassettes have the same or opposite orientation as the targeted genes, respectively

### Optimizing the insert insertion cassette

**Fig. 6:**
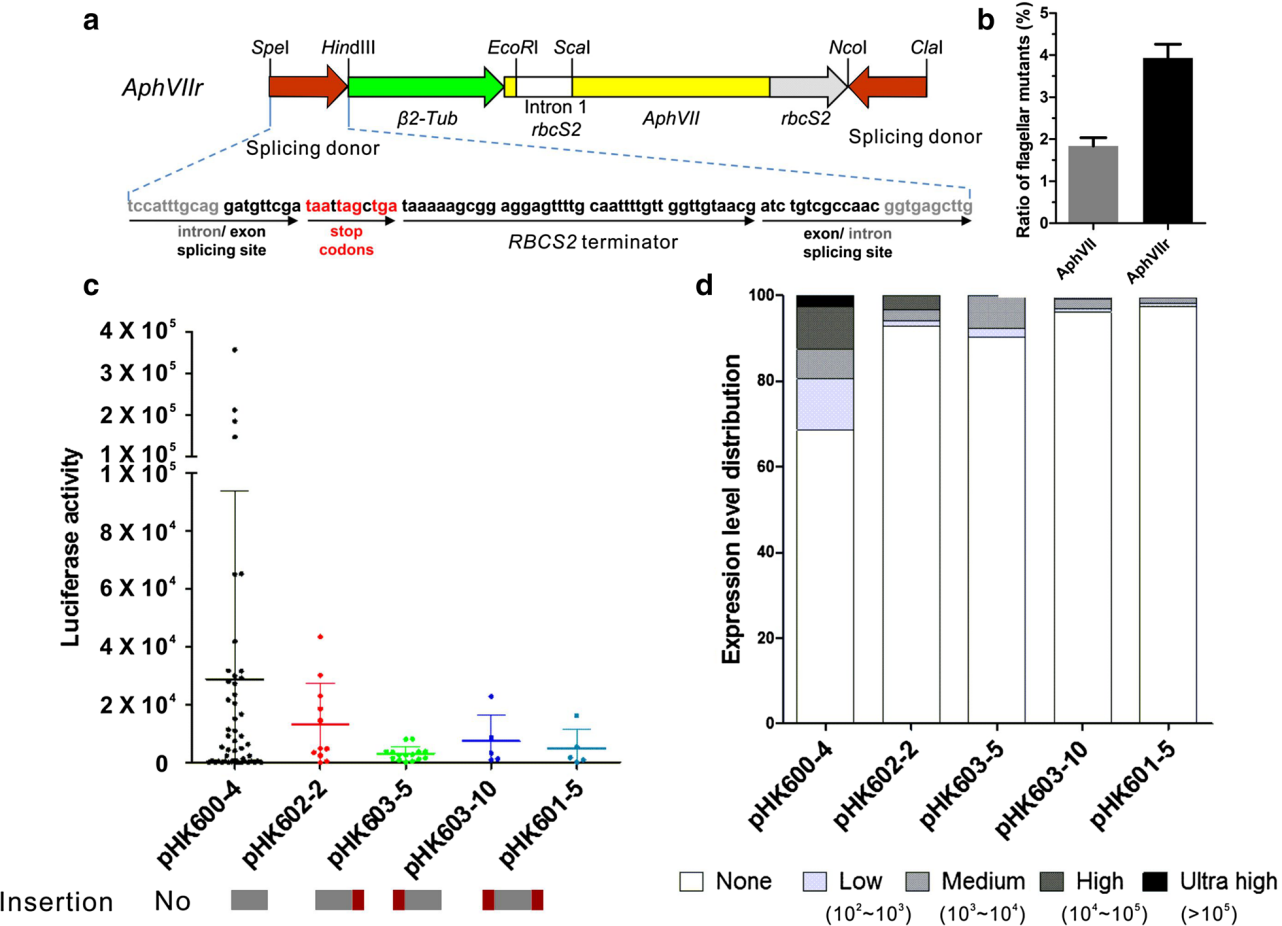
Generating a novel insertion cassette. **a** Diagram of insertion cassette *AphVIIr* which contains the 1.7-kb *AphVII* gene, forward splicing donor sequence, and reversed splicing donor sequence. Splicing donor sequence is indicated below. Intron/exon splicing sites, stop codons, and terminator are marked. **b** The *AphVIIr* insertion cassette generated more than 2 times motility-defect mutants as compared to insertion cassette *AphVIII*. **c** The luciferase activity of *Chlamydomonas* cells transformed with different insertion cassettes. Luciferase interrupted by insertion cassettes possessing splicing donor sequence displayed relatively lower activity. The pattern of splicing donor sequence (*red bar*) is indicated by the side of *AphVII* (*gray bar*). The cutoff of positive luciferase activity is set as 100. **d** The distribution of luciferase expression level in *Chlamydomonas* transformants cells. Insertion cassettes possessing splicing donor sequence generated less transformants displaying positive luciferase expression

Although the 2.6-kb *Mly*I-digested fragment contains two inverted terminators at its right border, it lacks a terminator on the left side. Additionally, terminators may not be functional for the target gene if the insertion cassette was located within an intron. To overcome this, we designed a new insertion cassette with a splicing donor sequence from the RubisCO gene (TCCATTTGCAG/GATGTTCGA) at both sides of the insert fragment. Thus, the insertion cassette became a part of the mRNA even if it was integrated into an intron. We also added three tandem overlapping stop codons and a transcription termination signal at each side (Fig. 6a). As a result, transcription of a target gene would terminate at the artificial transcription termination signal and polyadenylated at the new terminal. Thus, transcription and translation would stop simultaneously. We transformed the modified *aphVIIr* fragment into the strain HS211, and found that more than twofold mutants with motility defects were generated under the same conditions as compared to the original *aphVII* fragment without splicing donor segments (Fig. 6b). We also simulated gene interruption by inserting different cassettes into the RbcS2 intron3 which was integrated to luciferase, and analyzed the luciferase activity of *Chlamydomonas* transformants in order to compare the termination efficiency of the new insertion cassette with or without splicing donor sequences (Additional file 4: Fig. S2). Luciferase interrupted by insertion cassettes possessing splicing donor sequence displayed relatively lower activity, with an average intensity of ~3000–7300 in pHK601-5, pHK603-5 and pHK603-10 compared to ~13,000 in cassettes pHK602-2 which has no splicing donor sequence (Fig. 6c). Furthermore, insertion cassettes with splicing donor sequence generated less positive luciferase transformants. Only 2.5 and 3.9% positive transformants could be detected in pHK601-5 and pHK603-10, compared to 7.2% in pHK602-2. Transformant displaying high luciferase activity (>10^4^) could be hardly detected in pHK601-5, pHK603-5 and pHK603-10 (Fig. 6d).

These results suggest that the new insertion cassette increases the efficiency of transcription termination, subsequently increasing the possibility of generating the null mutant.

## Discussion

Due to a lack of reliable targeted gene editing methods such as TALEN or CRISPR-Cas9, large-scale insertion mutagenesis provides an alternative strategy to screen mutants of specific genes in *Chlamydomonas*. In this study, we generated and maintained an insertional mutagenesis library including approximately 150,000 clones, which can be utilized for both, reverse and forward genetics. A total of 26 mutants corresponding to 20 interested genes, related to glycosylation, starch metabolism, lipid metabolism, and flagellar proteins were identified by PCR-based screening. Meanwhile, two independent libraries related to flagellar assembly and oil droplet formation were obtained from this insertional library based on phenotype screening. The flagellar assembly mutant library included 967 mutants, which was expected to cover most of the ~1000 flagellar proteins [52, 53].

One advantage of this insertional library is that we transformed a fluorescence-labeled marker IFT46::YFP into the parental strain, which enabled us to screen specific mutants with defects in IFT. We obtained three mutants with reduced IFT46 expression in whole cells but lacking the accumulation at the basal body. The insertion loci of these mutants were identified and the interrupted genes may be involved in transporting or anchoring the IFT complex to the basal body. We also optimized several procedures for library construction and screening. First, the parental strain HS211 not only contains fluorescence IFT marker but also displays higher transformation efficiency. We also noticed that a strain expressing the *aphVIII* gene showed 4.5-fold higher efficiency than wild type if another selection marker such as *aphVII* was used. This indicates that *Chlamydomonas* tends to acquire a second antibiotic resistance more easily if an antibiotic resistance gene is already expressed. The molecular mechanism of this phenomena still need to be identified, but it speeded library construction by reducing the number of transformation. Second, we optimized the insertion cassette specific primer, which complement to the junction between two inverted terminator, and increased the specificity for the PCR screening. Third, we maintained all the transformants on the 9-cm sized agar plate with a fixed 48-clones format. This format ensures much more cells to be maintained for replication and further analyses. Because there is more space between each colony, the cross contamination rates were reduced. However, the procedure of picking fresh transformants into 48-clones format is labor-intensive and cryopreservation of the whole library in liquid nitrogen is recommended to prevent mutant loss. Finally, we normalized the amount of genomic DNA of each transformant by picking the same amount of each individual clones from solid agar plates to make the DNA mixture, which increase the chance to identify the insertion junction of mutants with similar PCR efficiency.

Interestingly, we found that the insertion cassette was inserted in UTR region among 30% mutants. The expression level of target gene was significantly reduced, which may be useful to screen the mutant of lethal genes. In addition, 46% of the insertions were localized in an intron. Their effect on the expression of the targeted gene varied. Hochmal et al., reported mutant IM*crx*, in which an insertion cassette was integrated in the second intron, displayed the same phenotype as the miRNA knock-down strain. However, minor calredoxin (CRX) expression was still detectable in the mutant induced by high-intensity light [54]. For mutants such as, *poc2*-*1*, in which the insertion is in an intron, partial of mRNA expression was detected by PCR. And this phenomenon is also reported in the one-off library transformed with a shorter PCR-fragment [29]. It is possible that the entire insertion cassette is spliced with the flanking intron sequence at the exon/intron boundaries, which results in the expression of an entire mRNA at relatively lower efficiency. Therefore, the effect of insertion in an intron on the expression of the targeted gene should be determined case by case. In order to improve the knock out efficiency and reduce the mRNA expression of the targeted gene, we designed a new cassette with splicing donor sequence and tandem overlapping stop codon at both sides, which is supposed to terminate the gene transcription immediately when it was inserted in an intron or UTR region.

In summary, our insertional mutant library provides an alternative to obtain mutants of genes of interest for the *Chlamydomonas* research community. Additionally, the sub-library of flagella and oil droplet assembly mutants will serve as a useful resource to investigate the molecular mechanism of flagellar assembly and oil droplet formation.

## Conclusions

In this study, we described comprehensive and economic methods to build an indexed mutant library including ~150,000 *C. reinhardtii* insertional mutants, which can be utilized for both reverse and forward genetics analysis. Using PCR-based screening method, we obtained 26 individual mutants corresponding to 20 different genes. Then, we identified 967 motility-defect mutants, including 10 mutants lacking IFT accumulation at the basal body. We also obtained 929 mutants defective in oil droplet assembly after nitrogen deprivation. In addition, a new insertion cassette was constructed to terminate the transcription of the targeted gene when the insertion cassette was inserted in introns and UTR. This library provides versatile resources for the research community to obtain *Chlamydomonas* mutants of any genes or with specific phenotype.

## Methods

### Algal strains and growth conditions

The flagella-less *C. reinhardtii* mutant and the wild type CC125 were kindly provided by the *Chlamydomonas* Resource Center. HS211 was used as parental strain for library construction. HS211 is a rescue strain of mutant *ift46*-*1* obtained by transforming a linearized plasmid pHK266, which contains the *IFT46* full length fused to the 5′ end of *YFP* (see “DNA manipulation” for details). Unless otherwise indicated, algal cells were grown mixtrophically in liquid TAP medium or on solid TAP agar (1.5%) plate under continuous illumination (30 μE m^−2^ s^−1^) at 22 °C. For maintenance of the library, algal colonies were grown under weak illumination (5 μE m^−2^ s^−1^) at 20 °C. For motility defect and oil droplet defect screening, algal colonies were inoculated into liquid TAP with omission of NH_4_Cl.

### DNA manipulation

The universal plasmid pHK86 includes fused *GFP*::*YFP* sequences driven by the *PSAD* promoter, and the selectable marker gene *AphVIII* driven by *HSP70A* promoter [55]. For mutant *ift46* rescue, the genomic DNA of *IFT46* gene was amplified by PCR from the plasmid pGEM T Easy-*IFT46* (provided by Joel Rosenbaum) using primers IFT46-F and IFT46-R (all primers listed in Additional file 3: Table S2). After digestion with *Nde*I and *Eco*RV, the *IFT46* fragment was ligated to the 5′ side of *YFP* in plasmid pHK86, yielding the plasmid pHK214. The *AphVIII* in pHK214 were exchanged with the *AphVII* fragment amplified from the plasmid pHyg3 (provided by Wolfgang Mages), which yielded the plasmid pHK266. The plasmid pHK266 was linearized by *Kpn*I and used to rescue the mutant *ift46*.

For library transformation, the 2.6-kb insertion cassette of *AphVIII* was digested from the plasmid pMJ013b with *Mly*I [27].

For optimization of the insertion cassette, the forward-splicing donor (FSD) sequence was synthesized by annealing the oligonucleotides F-Spe-F, F-Spe-R, F-Hind-F, and F-Hind-R, and the reversed one (RSD) was synthesized by annealing the oligonucleotides R-Nco-F, R-Nco-R, R-Cla-F, and R-Cla-R. The annealing process followed was as previously described by Hu et al. [38] After digestion with *Spe*I/*Hin*dIII or *Nco*I/*Cla*I, the forward and reverse splicing donor sequences were ligated to the 5′- and 3′-ends of *AphVII*, respectively, using pBluescript II KS as the backbone, which yielded the plasmid pHK415. The 1.9-kb fragment *FSD::AphVII::RSD* digested from pHK415 by *Spe*I/*Cla*I was used for transformation.

The RbcS2 intron3 was ligated to the CTGGAG/GTGCTG of luciferase using In-Fusion kit (Clontech). Different version of insertion cassettes were amplified from pHK415 and ligated to the restriction site of *Apa*I in *RbcS2* intron3.

### Nuclear transformation of *Chlamydomonas reinhardtii*

Nuclear transformations of *C. reinhardtii* were performed by electroporation as previously described by Huang et al. [56]. In brief, algae cells were grown in 200 ml TAP medium until the cell density reached 6 × 10^6^ cells/ml. The cells were concentrated to 2 × 10^8^ cells/ml with pre-chilled TAP, supplemented with 60 mM Sorbitol. Then, 250 μl concentrated cells were mixed with 30 ng DNA fragment in a 4-mm electroporation gap cuvette at 4 °C. Electroporation was performed using ECM630 electro cell manipulator (BTX Harvard Apparatus) with following parameters: voltage, 800 V; resistor, 1575 Ω; and capacitor, 50 μF. Transformants were recovered by incubation in 10 ml liquid TAP (60 mM sorbitol) for 14 h under low illumination (5 μE m^−2^ s^−1^). The cells were sedimented by centrifugation at 2500 rpm for 3 min and the pellet was mixed with 1 ml 20% starch and positive colonies were screened on solid TAP agar plates containing 10 μg/ml paromomycin or 12.5 μg/ml hygromycin B.

### DNA and RNA extraction

Total genomic DNA of *Chlamydomonas* was extracted using standard CTAB/phenol/chloroform method as previously described by Huang et al. [48].The DNA concentration of each super library was determined using *Hin*dIII digested lambda DNA (ThermoFisher Scientific, USA) as control and diluted to 100 ng/μl for PCR. Total RNA of *Chlamydomonas* was isolated using TRIzol^®^ reagent (ThermoFisher Scientific, USA) according to the manufacturer protocol. RNA concentration was determined by Quawell 5000 (Quawell Technology, USA). Residual genomic DNA in total RNA was removed by DNase I (RNase-free) (ThermoFisher Scientific, USA) before reverse transcription.

### PCR procedures

For PCR-based screening, the target gene specific primers were designed using Primer Premier 5.0 software with following parameters: length, 20–26 bp; Tm, 58–62 °C; GC%, 40–60% (all primers listed in Additional file 3: Table S2). PCR was performed in a 20-μl solution including 0.25 μM target gene specific primer, 0.125 μM insertion cassette specific primer LGR06-F, 0.2 mM dNTP, 0.5 M betaine (Sigma), 3% DMSO (Amresco), 0.5 unit TransTaq DNA polymerase (HiFi) (Transgen, China) and 100 ng genomic DNA templates. The PCR program for super library screening was as follows, pre-incubation at 95 °C for 5 min, 34 cycles of denaturation at 95 °C for 30 s, annealing at 62 °C for 30 s, and amplification at 72 °C for 2 min. The colony PCR was performed according to the method previously described by Cao et al. [57].

For mRNA expression quantification, reverse transcription of RNA was performed with oligo-dT primers using First Strand cDNA Synthesis Kit (ThermoFisher Scientific, USA) according to the manufacturer instructions. Quantitative real-time PCR was performed by ABI 7900HT Fast Real-Time PCR System (Applied Biosystems) with SYBR Green Realtime PCR Master Mix (TOYOBO, Japan) according to the manufacturer instructions. Using housekeeping gene *Chlamydomonas* β subunit-like polypeptide (*CBLP*, Cre06.g278222) as an internal control [58], the expression of other genes was quantified using standard curve assay in SDS 2.4 software (Applied Biosystems). All primers used in real-time PCR are listed in Additional file 3: Table S2.

For flanking sequence identification, RESDA-PCR was employed [59]. Degenerated primers (DegAluI, DegPstI, DegSacII, and DegTaqI) and primer Q0 were the same as the original method. Primers For1, For2, Rev1, and Rev2 were used as specific primers.

### Protein extraction and western blot analyses

Whole-cell protein extraction and concentration were performed as previously described by Hu et al. [38] Whole cell proteins (5 μg) were separated by 10% sodium dodecyl sulfate–polyacrylamide gel electrophoresis and transferred to 0.45-μm nitrocellulose membranes (Bio-Rad, USA) at 300 mA for 3 h. After blocking with 5% non-fat milk, the nitrocellulose membranes were probed with rabbit anti-IFT46 antibody (1:5000, immunized with the *N*-terminal 20 amino-acid peptide by Genscript) or mouse anti-*α*-tubulin antibody (1:200,000, #T9026, Sigma). Immunodetection of IFT46 was detected by ECL (Millipore) with goat anti-rabbit secondary antibody (1:5000, #6154, Sigma), and *α*-tubulin was detected with goat anti-mouse secondary antibody (1:5000, #A4416, Sigma).

### Extraction of total cellular lipids and thin-layer chromatography

Extraction of total cellular lipids was performed as previously described by Bligh and Dyer [60] with minor modifications. In brief, cells (approximately 3 × 10^7^ cells) were harvested by centrifugation at 6000*g* at room temperature for 5 min. The pellets were mixed with 400 µl methanol:chloroform (1:1, v/v) and vortexed for 5 min. The suspension was mixed with 120 μl PAK buffer (1 M KCl, 0.2 M phosphate acid). The mixture was centrifuged at 12,000*g* for 5 min and the lower chloroform phase was transferred to a new glass tube. Finally, additional chloroform was added into the organic phase until the constant volume was 20 µl.

For thin-layer chromatography, equal volume of total cellular lipid extract was loaded as a spot on 10 × 20 cm silica gel GF254 TLC plates (Haiyang, China). Neutral lipids in the samples were separated using a hexane/diethylether (3/1, v/v) solvent mixture. Then, the silica gel plates were dried under the hood and stained by iodine vapor at 37 °C for 10 min. Finally, the triacylglycerol bands were visualized by CanoScan 9000F (Canon, Japan) and analyzed by Quantity One software. All data were normalized to positive control.

### Microscopy

Motility-deficient mutants were identified using stereomicroscopy (Nikon SMZ1500, Japan). Mutants with changes in IFT46::YFP basal body localization were screened using LSM710 confocal microscope (Carl Zeiss, Germany) (excitation: 514 nm; emission: 519–560 nm). To screen oil droplet accumulation-deficient mutants, cells were stained in 0.1 μg/ml Nile red (Sigma, USA) for 15 min in the dark at 37 °C [61]. Cells with fewer oil droplets were identified using ECLIPSE Ti-U fluorescence microscope (Nikon, Japan) (excitation: 465–495 nm; dichroic mirror: 505 nm; emission: 515–555 nm).

Live cell images were captured using Leica SP8 confocal microscope. For Nile red, the excitation was 488 nm and the emission was 560–590 nm. For chlorophyll fluorescence (CHL), the excitation was 488 nm and the emission was 650–700 nm.

### Measurement of flagellar length

*Chlamydomonas* cells were fixed in 0.5% Lugol’ s iodine solution and examined with differential interference contrast (DIC) microscopy using ECLIPSE Ti-U fluorescence microscope (Nikon, Japan) equipped with a 40× objective. Lengths of flagella were measured with NIS-Elements BR software. For each sample, at least 300 flagella were measured.

### Luciferase activity assay

The luciferase activity was analyzed using the Renilla Luciferase Assay System (Promega, TM055) in 96 well microtiter plates according to the manual, with the modification that the algae cells were ruptured with glass-beads by vortex for 15 min.

## Additional files

**Additional file 1: Figure S1.** Comparison of the specificity of primer LGL03 and LGR06. More than sixteen non-specific PCR products were obtained with primer LGL03, whereas only four non-specific bands were obtained using primer LGR06.

**Additional file 2: Table S1.** List of genes for insertional mutants screening and PCR identified insertion loci from super libraries.

**Additional file 3: Table S2.** List of primers used in plasmid construction, RESDA-PCR, Realtime-PCR, and library screening.

**Additional file 4: Figure S2.** Different version of insertion cassettes. The 238 bp intron3 of *RbcS2* was fused to the luciferase at the site of CTGGAG/GTGCTG. The insertion cassette with or without splicing donor sequence at either side of *AphVII* gene was inserted to the restriction site of *Apa*I in *RbcS2* intron3.

## Abbreviations

CRISPR-Cas9: clustered regularly interspaced short palindromic repeats and CRISPR-associated protein 9
IFT: intraflagellar transport
EMS: ethyl methanesulfonate
MNNG: *N*-methyl-*N*′-nitro-*N*-nitrosoguanidine
HR: homologous recombination
JGI: joint genome institute
RESDA-PCR: restriction enzyme site-directed amplification
TAP-N: nitrogen-deprived TAP medium
TLC: thin layer chromatography
TAG: triacylglycerol
FSD: forward-splicing donor
RSD: reverse-splicing donor
