# Supplementary figures and images for: Building a multipurpose insertional mutant library for forward and reverse genetics in Chlamydomonas
Source: Plant Methods. 2017 May 15;13:36. doi: 10.1186/s13007-017-0183-5 (PMC5430608; doi:10.1186/s13007-017-0183-5)

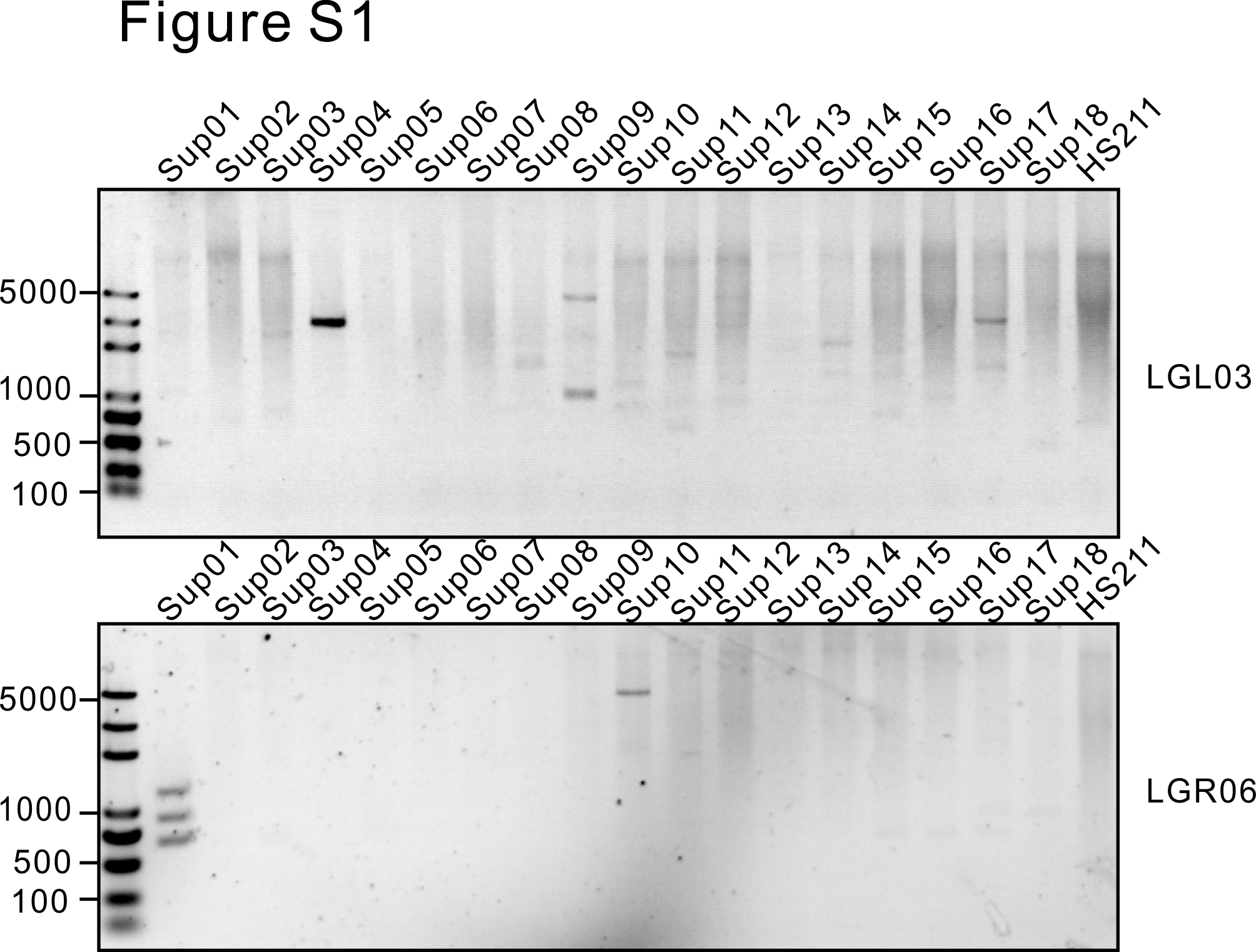

Supplement: Supplementary file 1 — Additional file 1: Figure S1. Comparison of the specificity of primer LGL03 and LGR06. More than sixteen non-specific PCR products were obtained with primer LGL03, whereas only four non-specific bands were obtained using primer LGR06. [file 13007_2017_183_MOESM1_ESM.tif]

pHK600-4

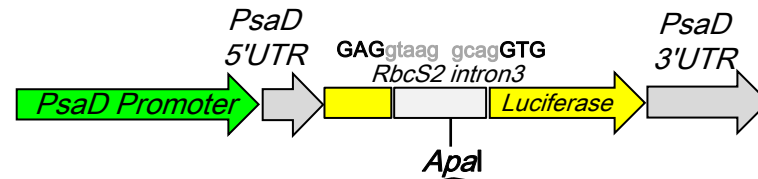

pHK601-5

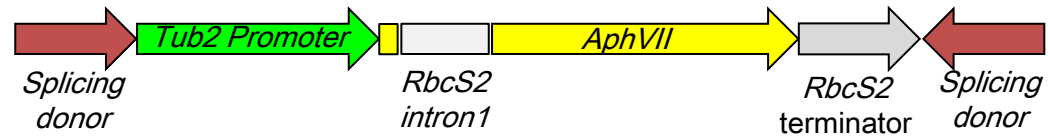

pHK602-2

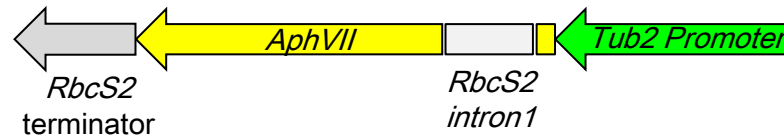

pHK603-5

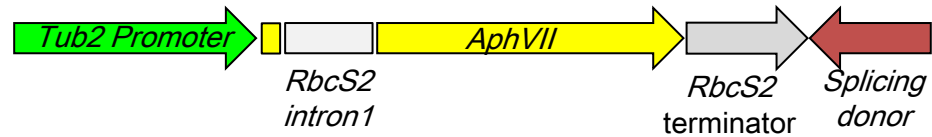

pHK603-10

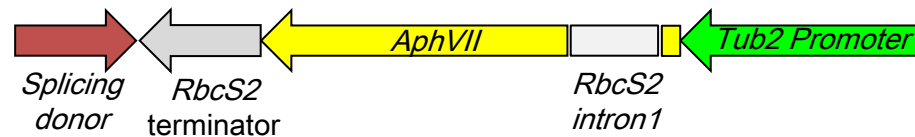

Supplement: Supplementary file 4 — Additional file 4: Figure S2. Different version of insertion cassettes. The 238 bp intron3 of RbcS2 was fused to the luciferase at the site of CTGGAG/GTGCTG. The insertion cassette with or without splicing donor sequence at either side of AphVII gene was inserted to the restriction site of ApaI in RbcS2 intron3. [file 13007_2017_183_MOESM4_ESM.pdf]
